# Predicting responsiveness to GLP-1 pathway drugs using real-world data

**DOI:** 10.1186/s12902-024-01798-9

**Published:** 2024-12-18

**Authors:** Xiaodong Zhu, Michael J. Fowler, Quinn S. Wells, John M. Stafford, Maureen Gannon

**Affiliations:** 1 Department of Veterans Affairs, Tennessee Valley Health Authority, Nashville, TN USA; 2 Department of Medicine, Vanderbilt University Medical Center, Nashville, TN USA; 3 Department of Biomedical Informatics, Vanderbilt University Medical Center, Nashville, TN USA; 4 Department of Molecular Physiology and Biophysics, Vanderbilt University, Nashville, TN USA; 5 Department of Cell and Developmental Biology, Vanderbilt University, Nashville, TN USA

**Keywords:** Electronic health record, GLP-1, Type 2 diabetes, Predictive model, HbA1c

## Abstract

**Background:**

Medications targeting the glucagon-like peptide-1 (GLP-1) pathway are an important therapeutic class currently used for the treatment of Type 2 diabetes (T2D). However, there is not enough known about which subgroups of patients would receive the most benefit from these medications.

**Objective:**

The goal of this study was to develop a predictive model for patient responsiveness to medications, here collectively called GLP-1 M, that include GLP-1 receptor agonists and dipeptidyl peptidase-4 (DPP4) inhibitors (that normally degrade endogenously-produced GLP-1). Such a model could guide clinicians to consider certain patient characteristics when prescribing second line medications for T2D.

**Methods:**

We analyzed de-identified electronic health records of 7856 subjects with T2D treated with GLP-1 M drugs at Vanderbilt University Medical Center from 2003–2019. Using common clinical features (including commonly ordered lab tests, demographic information, other T2D medications, and diabetes-associated complications), we compared four different models: logistic regression, LightGBM, artificial neural network (ANN), and support vector classifier (SVC).

**Results:**

Our analysis revealed that the traditional logistic regression model outperforms the other machine learning models, with an area under the Receiver Operating Characteristic curve (auROC) of 0.77.Our model showed that higher pre-treatment HbA1C is a dominant feature for predicting better response to GLP-1 M, while features such as use of thiazolidinediones or sulfonylureas is correlated with poorer response to GLP-1 M, as assessed by lowering of hemoglobin A1C (HbA1C), a standard marker of glycated hemoglobin used for assessing glycemic control in individuals with diabetes. Among female subjects under 40 taking GLP-1 M, the simultaneous use of non-steroidal anti-inflammatory drugs (NSAIDs) was associated with a greater reduction in HbA1C (0.82 ± 1.72% vs 0.28 ± 1.70%, *p* = 0.008).

**Conclusion:**

These findings indicate a thorough analysis of real-world electronic health records could reveal new information to improve treatment decisions for the treatment of T2D. The predictive model developed in this study highlights the importance of considering individual patient characteristics and medication interactions when prescribing GLP-1 M drugs.

**Supplementary Information:**

The online version contains supplementary material available at 10.1186/s12902-024-01798-9.

## Key points

1. Patient characteristics such as poorer blood glucose control, higher body mass, and shorter duration of diabetes predict better response to medications that target the GLP-1 pathway.

2. Simultaneous use of NSAIDs (for example ibuprofen) was associated with better responsiveness in women under 40.

3. Combining GLP-1 pathway medications with some other commonly used T2D medications (for example thiazolidinediones or sulfonylureas) may not have an additional benefit.

## Introduction

Over 460 million people worldwide have diabetes [1]. In the United States, 10.5% of the population has diabetes, with about 90–95% of those having T2D [2]. Metformin is the preferred first-line treatment for.

T2D. However, due to the progressive nature of diabetes, a second-line medication is commonly required at some point in the course of the disease to maintain glucose homeostasis. When combined with metformin, on average all non-insulin medications lower hemoglobin A1C (HbA1C) by about 0.7–1.0% [3]. However, at the individual level, patients are known to respond differently to different medications [4]. Moreover, many factors contribute to diabetes etiology and responsiveness to medications, including patient characteristics and environmental factors [5]. Unfortunately, there are few widely used biomarkers to aid in predicting individual patient responses [6]. Therefore, it is important to understand real-world patient responses to different combinations of medications to enhance personalized treatment for T2D. 

When cost is not an issue, the ADA recommends choosing a second-line medication from one of the following: dipeptidyl peptidase-4 inhibitors (DPP4i), glucagon-like peptide-1 receptor agonists (GLP-1-RA), or sodium/glucose co-transporter inhibitors (SGLT2i) [3]. Among these options, the incretin-based drugs GLP-1-RA and DPP4i (which we will collectively call GLP-1 M) are widely used. From 2013 to 2016, 23% of T2D patients in the US received DPP4i, and 6% of patients received GLP-1-RA [7]. A recent recommendation published by a group of diabetologists suggests that GLP-1-RA are currently under-prescribed [8], and thus, use of GLP-1 M is likely to continue to increase in the future. GLP-1-RA mimic the actions of GLP-1, binding the GLP-1 receptor on the surface of β cells (and other cell types) and stimulating insulin secretion in the presence of elevated glucose. DPP4i block the activity of the enzyme that normally degrades endogenously produced GLP-1, thus, increasing its half-life in the circulation.

Importantly, despite their wide-spread usage, patient responses to GLP-1 M are heterogeneous. A study that analyzed the effects of GLP-1-RA demonstrated that patients with baseline HbA1C of 7–8% had only a 40%−60% probability of reducing HbA1C to < 7%, after 12-months of GLP-1 M treatment [9]. In addition to differences in patient genetics and pathophysiology, drug-drug interactions could contribute to the heterogenous response to GLP-1 M. GLP-1 M act through a G protein coupled receptor (GPCR) that couples to Gαs. Islets express nearly 300 GPCRs [10] and GPCRs represent 35% of all current drug targets [11]. It is highly possible that activity of other GPCRs, and medications that affect their signaling, modulate GLP-1 M activity in β cells.

In this study, we aimed to develop a predictive model for patient responsiveness to GLP-1 M treatment. We analyzed the de-identified electronic health record (EHR) database entries from 2003–2019 at Vanderbilt University Medical Center (VUMC). Subject demographics (for example: age, sex, self-identified race), lab values (for example: HbA1c, BMI, liver enzymes), and some medication information (GLP-1 M, other drugs used to treat T2D, and NSAIDs) and presence or absence of diabetes-associated complications (for example: chronic kidney disease, cardiovascular disease) were extracted as prediction features. We compared logistic regression, LightGBM, artificial neural networks (ANNs), and support vector classifier (SVC), to identify the best model for predicting GLP-1 M responsiveness. We found logistic regression to be the best model. Pre-treatment HbA1C was the most important prediction factor for responsiveness. We also found associations between usage of other T2D medications or NSAIDs with GLP-1 M responsiveness.

## Methods

### Data source and ethics

This study was approved by the VUMC Institutional Review Board (IRB; #191,602) in Nashville, TN. All data are from the VUMC Synthetic Derivative (SD) database, a de-identified copy of the main hospital medical record database created for research purposes. The SD contains over 3.2 million electronic records, with no defined exclusions. No HIPAA identifiers are available in the SD. Dates, such as “January 1, 2004” have been replaced with a randomly generated date, such as “February 3, 2003”. To maintain data integrity for each subject, a single random time interval was generated, and then used to shift all the dates for the same subject. In this way, all the time intervals of subject clinic visits and treatments remain intact.

### Defining the subject cohort for analysis

In this study, ICD codes were used to identify the disease. The International Classification of Diseases (ICD) 9 and ICD10 codes used to define T2D are listed in Supplemental Table 1. All other ICD and CPT (Current Procedural Terminology) codes used to identify the other diseases and procedures are listed in Supplemental Table 2. To be included, each diagnosis required that the ICD code be present a minimum of twice in the subject’s record.

**Table 1:** Mean values ± SD of subjects’ continuous features considered

**Features**	**Number of subjects**	**Mean value ± SD**
HbA1c decrease	7856	0.50 ± 1.57
HbA1C before treatment	7856	8.01 ± 1.72
HDL	5635	43.35 ± 12.63
LDL	5279	105.33 ± 74.50
Triglycerides	5652	209.66 ± 162.08
Total cholesterol	5713	176.45 ± 45.89
SGOT (AST)	6480	29.24 ± 20.07
SGPT (ALT)	6546	32.80 ± 25.90
C -peptide	324	4.05 ± 3.22
Vitamin B12	1094	747.69 ± 926.74
Blood creatinine	7191	1.07 ± 0.70
Fasting blood glucose	1215	164.56 ± 62.13
Random glucose	5922	174.97 ± 72.13
Urine glucose	1745	30.97 ± 209.83
DBP	7769	76.12 ± 9.99
SBP	7769	132.90 ± 14.72
BMI	7705	35.45 ± 8.07
Blood albumin	6168	4.19 ± 0.36
Albumin /creatinine ratio	4019	72.63 ± 265.25
Urine albumin	4021	73.97 ± 256.83
Bicarbonate	399	25.20 ± 5.29
Total blood protein	3478	7.25 ± 0.52
Blood calcium	7087	9.53 ± 0.48
Blood chloride	7089	102.34 ± 3.36
Vitamin D 25OH	1215	29.93 ± 14.05
Whole blood lactate	265	1.50 ± 0.77
O2 saturation	256	97.68 ± 3.10
Blood potassium	7103	4.23 ± 0.42
Pulse	6895	80.03 ± 12.06
Respiration rate	3838	17.48 ± 3.43
Blood sodium	7097	138.54 ± 2.59
Age	7856	57.30 ± 11.90
T2D duration	7856	3.36 ± 3.95

Mean values of subjects’ features used in the current anaylsis. Continuous features

**Table 2:** Mean values ± SD of subjects’ discrete features considered

**Features**	**Number of subjects (% of total)**
Total subjects	7856
Female	3911(49.78%)
Not Hispanic	7635(97.19%)
Africa American	1386(17.64%)
White	6193(78.83%)
Asian	149(1.90%)
Other	147(1.87%)
Chronic kidney disease	1069(13.61%)
Cardiomyopathy	341(4.34%)
Heart failure	654(8.32%)
Hypertension	5566(70.85%)
Arthritis	1447(18.42%)
Gastric bypass	148(1.88%)
Bowel resection	82(1.04%)
Retinopathy	333(4.24%)
Insulin	2031(25.85%)
Metformin	4734(60.21%)
Sulfonylureas	3041(38.71%)
Thiazolidinediones	773(9.84%)
NSAIDs	3966(50.48%)
Painkiller	1542(19.63%)
Other T2D medication	488(6.21%)
Smoking	2888(36.76%)

Mean values of subjects’ features used in the current anaylsis. Binary/discrete features

**Fig. 1:**
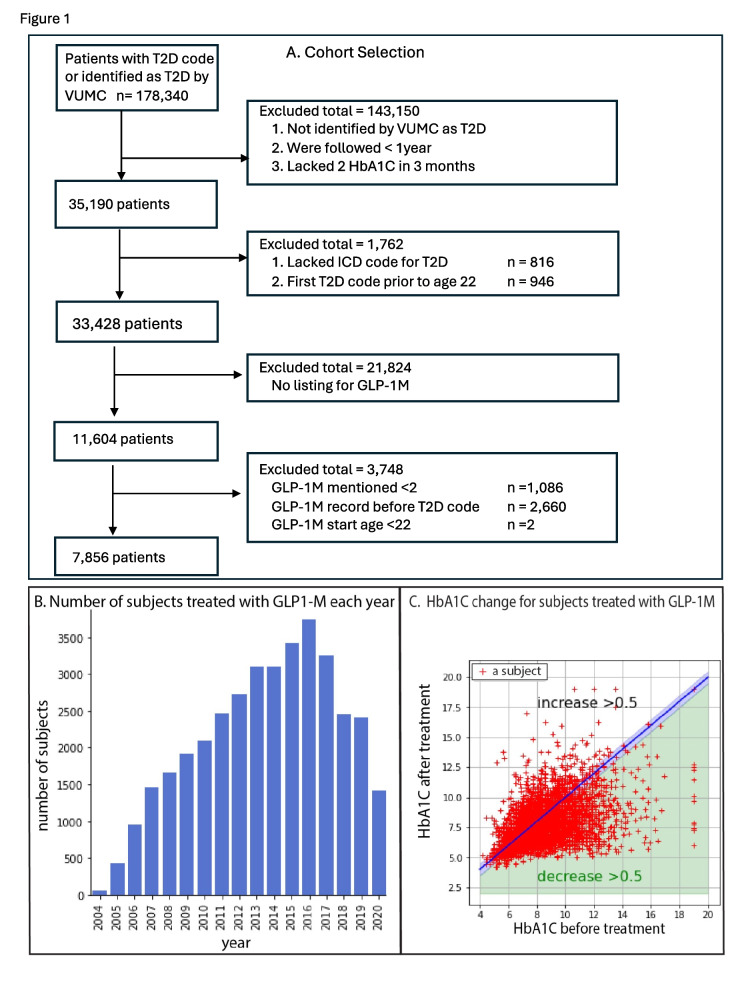
Cohort inclusion and exclusion strategy and response to GLP-1 M. **A**. Overview of the cohort building procedure. **B**. The number of subjects treated with GLP-1 M per year during the defined period (see Methods) in the VUMC Synthetic Derivative database. The years indicated are synthetic time (see Methods). **C**. Subject responsiveness to GLP-1 M is heterogeneous. Each ‘ + ’ indicates one subject. The blue line indicates an unchanged HbA1C level after GLP-1 M treatment. The blue shading contains subjects whose HbA1C levels were changed by less than ± 0.5% in the treatment period. The green shading contains subjects whose HbA1C decreased by more than 0.5% in the period after treatment. White area contains subjects whose HbA1C increased by 0.5% or more in the time period after treatment

At the time of our analysis, there were 178,340 subjects in the SD with at least one ICD code for T2D (see Supplemental Table 1). Figure 1A shows the strategy used for cohort selection. Subjects with T2D were identified using a published algorithm based on lab tests, prescriptions, and ICD codes [12]. This algorithm was previously validated across five institutions and demonstrated a positive predictive value (PPV) of 98%. We further restricted our analysis to subjects with T2D whose medical records also met the following requirements: 1. included at least two HbA1C measurements spanning at least one year; 2. included at least one ICD code for T2D (Supplemental Table 1); and 3. the first T2D code occurred after age 22 years. These criteria identified 33,428 subjects with T2D. Among these, one-third (11,604 subjects) had at least one record of GLP-1 M treatment. We considered a subject as having been treated with GLP-1 M only if there were at least two different medication entries referring to GLP-1 M treatment in the subject record, and GLP-1 M treatment occurred together with or subsequent to ICD codes for T2D (10,517 subjects were selected using these rules). Among these, 7,856 subjects had HbA1C values both before and after GLP-1 M treatment; this was the final number of subjects included in the analysis.

Each of the FDA-approved GLP-1Ms have been prescribed at VUMC (Supplemental Table 3), although the prevalence for each varied. Among this class of drugs, albiglutide (US. trade name: Tanzeum) was found to be less effective than other drugs in this class and was withdrawn from the market in 2018. We therefore excluded albiglutide use from our analysis. It should be noted that the first record of GLP-1 M usage in the VUMC database was in 2004 (Fig. 1B). However, the first GLP-1 M, ByettaTM (Exenatide), was actually approved in 2005. The discrepancy in the dates is due to the fact that for each subject, all the dates in the VUMC SD database have been randomly shifted backward by 0–364 days. All the dates in our study therefore refer to these “synthetic dates”. The prescribing of GLP-1 M In VUMC increased consistently until 2017 (Fig. 1B), and then decreased. Multiple factors may contribute to the variation of the usage of the drug. The underlying reason for this pattern of the drug application is beyond the scope of the current paper.

**Table 3:** Differences in feature values between GLP-1 M responders and non-responders

**Features**	**Number of responders**	**Responder value mean** ± SD	**Number of non-responders**	**Non-responder value** **mean** ± SD	**False Discovery Rate** (**FDR)**
HbA1C before treatment	3447	8.87 ± 1.84	4409	7.34 ± 1.27	** < 1.0E-100**
HDL cholesterol	2467	42.52 ± 12.19	3168	44.00 ± 12.92	**1.04E-05**
LDL cholesterol	2282	108.66 ± 78.45	2997	102.79 ± 71.26	**3.33E-04**
Triglycerides	2477	222.99 ± 176.99	3175	199.27 ± 148.61	**9.54E-08**
Total cholesterol	2503	179.62 ± 47.28	3210	173.98 ± 44.62	**9.08E-06**
SGOT (AST)	2838	30.56 ± 22.40	3642	28.21 ± 17.99	**1.71E-06**
SGPT (ALT)	2872	35.08 ± 26.65	3674	31.01 ± 25.16	**1.81E-18**
C-peptide	193	4.03 ± 3.25	131	4.07 ± 3.19	4.52E-01
Vitamin B12	455	792.49 ± 998.19	639	715.79 ± 871.71	5.85E-02
Blood creatinine	3169	1.03 ± 0.59	4022	1.10 ± 0.77	**1.87E-05**
Fasting blood glucose	512	177.76 ± 69.58	703	154.94 ± 54.14	**4.89E-09**
Random glucose	2618	197.21 ± 80.67	3304	157.34 ± 58.88	**5.11E-105**
Urine glucose	708	64.80 ± 251.71	1037	7.87 ± 171.95	**2.52E-09**
DBP	3418	76.79 ± 9.96	4351	75.60 ± 9.99	**1.71E-06**
SBP	3418	133.42 ± 14.59	4351	132.48 ± 14.82	**4.06E-03**
BMI	3390	35.77 ± 7.95	4315	35.19 ± 8.16	**1.14E-04**
Blood albumin	2706	4.19 ± 0.36	3462	4.18 ± 0.36	3.45E-01
Albumin creatinine ratio	1804	75.81 ± 257.71	2215	70.05 ± 271.26	**6.24E-08**
Urine albumin	1804	80.73 ± 263.27	2217	68.48 ± 251.40	**7.41E-08**
Bicarbonate	157	25.10 ± 5.33	242	25.27 ± 5.28	3.44E-01
Total blood protein	1470	7.26 ± 0.51	2008	7.24 ± 0.51	6.58E-02
Blood calcium	3123	9.54 ± 0.47	3964	9.51 ± 0.48	**5.63E-03**
Blood chloride	3123	101.82 ± 3.37	3966	102.74 ± 3.29	**1.21E-29**
Vitamin D 25OH	498	29.03 ± 13.23	717	30.56 ± 14.56	1.04E-01
Whole blood lactate	94	1.51 ± 0.70	171	1.50 ± 0.81	2.91E-01
O2 saturation	101	97.39 ± 3.76	155	97.87 ± 2.58	3.45E-01
Blood potassium	3131	4.23 ± 0.41	3972	4.24 ± 0.42	1.60E-01
Pulse	2973	81.07 ± 12.26	3922	79.25 ± 11.85	**1.14E-08**
Respiration rate	1640	17.49 ± 3.15	2198	17.47 ± 3.62	9.85E-02
Blood sodium	3127	138.13 ± 2.64	3970	138.87 ± 2.50	**4.29E-33**
Age	3447	56.61 ± 11.68	4409	57.83 ± 12.04	**1.71E-06**
T2D duration	3447	3.37 ± 3.98	4409	3.36 ± 3.93	3.31E-01

Comparison of features of GLP-1M responders versus non-responders. Significant values are shown in bold in the right hand column

### Data pre-processing

For each type of lab value, the data was first converted to the same unit. A range was then used to identify and eliminate likely incorrectly entered values that fell outside known published value ranges for that particular test. For blood pressure values, when the diastolic pressure was normal, but systolic pressure was below 20, we multiplied the systolic pressure with a correction factor of 10. We developed this correction since a systolic pressure below 20 mm Hg is not compatible with life, and the presence of a normal diastolic pressure value in the record further supports that the extremely low systolic value is an error. Such an error is most likely due to the omission of a trailing zero. We did not perform corrections for other values, as it is difficult to distinguish between genuine data points and errors.

If one subject had multiple records for the same lab test, we determined the median value, as it is robust to the outlier. For the other categorical features, we used binary encoding. In our dataset, race was not mutually exclusive, e.g., a subject can have the value 1 for being both White and Asian. Race is self-reported by subjects and thus, if a subject listed two races on their intake survey, we could not exclude one or the other, since all subjects are deidentified. Only a very small percentage of our subjects have a self-reported mixed racial background and reflects real-world diversity. Excluding them from the study could lead to racial bias, so we retained them in our dataset.

For HbA1C, the SD contains HbA1C measurements obtained via the VUMC clinical laboratory as well as point of care (POC) testing. There is only an average of 0.2% difference between the average of the two values (Supplemental Fig. 1A). Moreover, no significant difference in pattern was observed when plotting these two values together (Supplemental Fig. 1B). Thus, we combined the HbA1C from these two different sources without any adjustment.

As our data span over 17 years, for each feature, after data processing, we examined the data range and distribution in each year, to ensure that the data could be combined. We did not identify any difference in data distribution, so no further adjustment was done. With the cleaned dataset, for each lab test, we used the median value within 12 months prior to initiation of GLP1-M treatment as the final value. For each diagnosis and surgery, we only considered it in our analysis if it occurred before initiation of GLP-1 M. We required any additional medications to be administered within ± 3 days of GLP-1 M treatment to be considered simultaneous.

### Response label creation

The binary label for treatment responsiveness was created by the following method: the median HbA1C level within 12 months before GLP-1 M treatment was used as the pretreatment HbA1C. The median HbA1C recorded 12 months after treatment was initiated was used as the after-treatment HbA1C. If the difference between the two values was more than 0.5%, we considered the subject to have responded to the treatment (score of 1). If not, the subject was designated as not responding (score of 0).

### Data analysis and model development

Python libraries such as Pandas, Numpy, Scipy, Sklearn, LightGBM, Keras, and Seaborn were used for data process and modeling. For tests with multiple entries, if the number of tests was relatively small, we used the Bonferroni Correction and present the adjusted p-value. If the number of tests was large, we computed the False Discovery Rate (FDR) to adjust for the multiple tests.

The percentage of missing values is shown in Supplemental Table 4. To build the model, only features with less than 30% of subject data missing were used. For logistic regression, ANN, and SVC, we applied the K-Nearest Neighbor (KNN) algorithm to fill in any missing values. Briefly, the missing value for each subject was replaced with the average value from the five most similar subjects calculated using Euclidian distance. We also used L1 regularization to generate a model with the least number of features. Since LightGBM is a gradient boost tree model that can model complex decision boundaries, it has no linear assumptions and can tolerate missing values. Thus, when using LightGBM, any missing values were not filled.

**Table 4:** Decrease in HbA1c in response to GLP-1 M based on subject features

**Features**		**Number of subjects**	**Decrease in HbA1c** **(mean** ± SD)	**False Discovery Rate** (**FDR)**
Sex	Female	3911	0.45 ± 1.48	**3.09E-02**
	Male	3945	0.55 ± 1.64	
Hispanic ethnicity	No	7635	0.50 ± 1.56	2.39E-01
	Yes	221	0.46 ± 1.58	
Race	Africa American	1386	0.55 ± 1.83	8.91E-02
	Non-African American	6470	0.48 ± 1.50	
	White	6193	0.49 ± 1.51	1.56E-01
	Non-White	1663	0.53 ± 1.75	
	Asian	149	0.19 ± 1.08	**1.06E-02**
	Non-Asian	7707	0.50 ± 1.58	
	Other	147	0.61 ± 1.34	9.96E-02
	Non-Other	7709	0.49 ± 1.57	
Chronic kidney disease	Yes	1069	0.32 ± 1.50	**6.65E-04**
	No	6787	0.53 ± 1.58	
Cardiomyopathy	Yes	341	0.32 ± 1.44	**3.19E-02**
	No	7515	0.51 ± 1.57	
Heart failure	Yes	654	0.33 ± 1.51	**4.58E-03**
	No	7202	0.51 ± 1.57	
Hypertension	Yes	5566	0.51 ± 1.51	**3.59E-03**
	No	2290	0.47 ± 1.69	
Arthritis	Yes	1447	0.45 ± 1.36	2.30E-01
	No	6409	0.51 ± 1.61	
Gastric bypass	Yes	148	0.64 ± 1.49	5.61E-02
	No	7708	0.49 ± 1.57	
Bowel resection	Yes	82	0.25 ± 1.55	2.23E-01
	No	7774	0.50 ± 1.57	
Retinopathy	Yes	333	0.28 ± 1.49	**3.96E-02**
	No	7523	0.51 ± 1.57	
Insulin	Yes	2031	0.66 ± 1.81	** < 1.0E-100**
	No	5825	0.44 ± 1.47	
Metformin	Yes	4734	0.54 ± 1.59	**1.78E-08**
	No	3122	0.43 ± 1.52	
Sulfonylureas	Yes	3041	0.49 ± 1.58	**5.16E-03**
	No	4815	0.50 ± 1.56	
Thiazolidinediones	Yes	773	0.37 ± 1.53	**3.29E-01**
	No	7083	0.51 ± 1.57	
NSAIDs	Yes	3966	0.46 ± 1.53	**5.16E-03**
	No	3890	0.54 ± 1.60	
Painkiller	Yes	1542	0.43 ± 1.56	**6.76E-03**
	No	6314	0.51 ± 1.57	
Other T2D Medication	Yes	488	0.49 ± 1.38	**6.08E-02**
	No	7368	0.50 ± 1.58	
Smoking	Yes	2888	0.53 ± 1.59	**2.82E-01**
	No	4968	0.48 ± 1.55	

Comparison of features of GLP-1M responders versus non-responders. Significant values are shown in bold in the right hand column

**Fig. 2:**
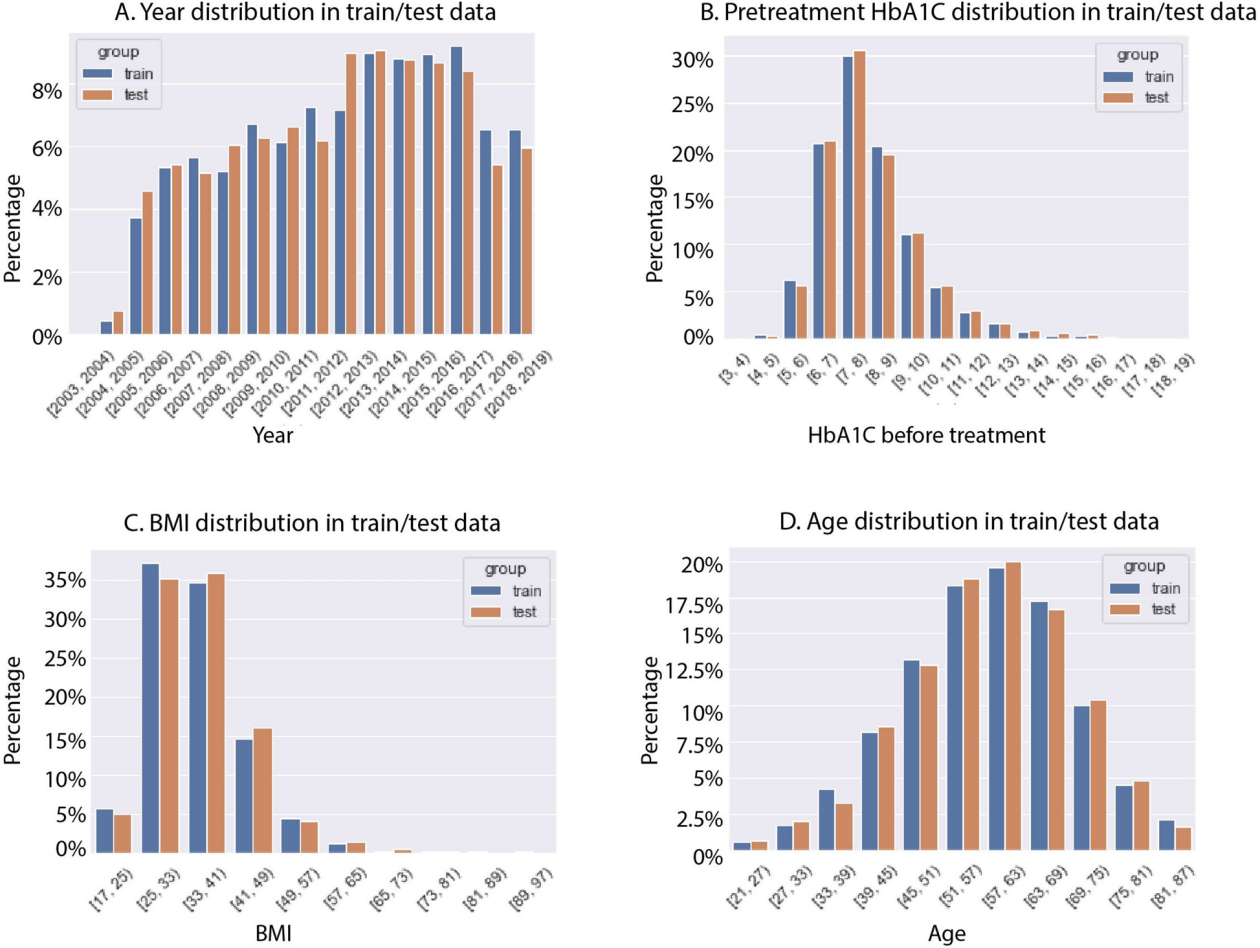
Histograms of training and testing data. Training data (blue) and testing data (orange) are similar for (**A**) treatment year and (**B**) HbA1C before treatment. (**C**) Cohort BMI. (**D**) Cohort age

The data were divided into a training set and testing set (Supplemental Table 5). Eighty-five percent of the data was used for model training and validation; 15% of the data was used for final testing. The data distribution was similar between both sets (Fig. 2) and we ensured that the distribution of treatment years was similar between training and testing data sets (Fig. 2A). The treatment time was not used for the training set to avoid data leakage. Grid search and five-fold cross-validation were then used to search for hyperparameters. During grid search, 90% of the training data was used for the model training set and 10% was used for validation.

**Table 5:** Differences in responder vs non-responder subjects (binary variables)

**Features**		**Number of responders (% of total)**	**False Discovery Rate** (**FDR)**
Sex	Female	1674(42.8%)	9.70E-02
	Male	1773(44.9%)	
Hispanic ethnicity	No	3349(43.8%)	9.42E-01
	Yes	98(44.3%)	
Race	Africa American	648(46.8%)	**3.41E-02**
	Non-Africa American	2799(43.3%)	
	White	2688(43.4%)	1.60E-01
	Non-White	759(45.6%)	
	Asian	46(30.8%)	**4.02E-03**
	Non-Asian	3401(44.1%)	
	Other	74(50.3%)	1.88E-01
	Non-Other	3373(43.7%)	
Chronic kidney disease	Yes	425(39.7%)	**8.05E-03**
	No	3022(44.5%)	
Cardiomyopathy	Yes	137(40.1%)	2.35E-01
	No	3310(44.0%)	
Heart failure	Yes	251(38.3%)	**7.58E-03**
	No	3196(44.3%)	
Hypertension	Yes	2494(44.8%)	**1.99E-02**
	No	953(41.6%)	
Arthritis	Yes	609(42.1%)	1.91E-01
	No	2838(44.3%)	
Gastric bypass	Yes	69(46.6%)	5.83E-01
	No	3378(43.8%)	
Bowel resection	Yes	33(40.2%)	6.01E-01
	No	3414(43.9%)	
Retinopathy	Yes	137(41.1%)	3.71E-01
	No	3310(44.0%)	
insulin	Yes	1017(50.1%)	**6.04E-10**
	No	2430(41.7%)	
Metformin	Yes	2130(44.5%)	**2.80E-02**
	No	1317(42.2%)	
Sulfonylureas	Yes	1359(44.7%)	3.29E-01
	No	2088(43.36%)	
Thiazolidinediones	Yes	289(37.3%)	**3.81E-04**
	No	3158(44.6%)	

Comparison of features of GLP-1M responders versus non-responders. Significant values are shown in bold in the right hand column

## Results

### Responses to GLP-1 M are heterogeneous

A meaningful decrease in HbA1C level is the most commonly used clinical measure for evaluating the effectiveness of any diabetes treatment. The median HbA1C level of all T2D subjects treated at VUMC was maintained at approximately 7.0 in the past 17 years, suggesting that overall, diabetes is well-controlled in this cohort.

A decrease in HbA1C of less than 0.5% has not been shown to correlate with improvements in the incidence of diabetic complications [13, 14]. Therefore, in this study, a subject was considered as a responder to GLP-1 M only when the HbA1C decreased by more than 0.5% after the treatment. We considered a subject to have a positive response to GLP-1 M if the median HbA1C within 12 months after the treatment decreased by more than 0.5% from the median HbA1C within 12 months before the treatment [13, 14]. Surprisingly, although at the population level HbA1C decreased by 0.50 ± 1.57% after GLP-1 M treatment (Table 1), a decrease in HbA1C of more than 0.5% was only observed in 43.9% of subjects (Fig. 1C, green area). In 36.7% of subjects, the HbA1C did not change by more than 0.5% in either direction, while in 17.7% of subjects, the HbA1C increased by more than 0.5% after GLP-1 M treatment (Fig. 1C, white area), potentially indicating a lack of response to drug treatment. It is important to note that GLP-1 M includes both DPP4i and GLP-1-RA, which have different mechanisms of action. DPP4i relies on endogenous GLP-1 to be effective, while GLP-1-RA does not. To investigate whether responses to these two medication categories differ, we examined patient responses to each medication separately (Supplemental Fig. 1C, D) and found a heterogeneous response to each drug, with a pattern similar to the analysis of the two treatments combined. As a result, we combined the two types of medications for the rest of the paper to increase the sample size.

Next to build a model predicting GLP-1 M treatment responsiveness, we retrieved demographic and clinical information (here, called features) for each subject. Since the data were collected as part of clinical care, features available for selection were limited to lab tests requested by the treating clinician. To have sufficient statistical power, we selected only features that were present in more than 100 subjects. Our final dataset for exploratory analysis, contained 34 continuous features (Table 1), including most of the commonly ordered lab tests, and 19 discrete features (Table 2), including demographic information, other T2D medications, and diabetes complications. The specific drug names can be found in Supplemental Table 3.

### Univariate analysis of features associated with GLP-1 M treatment effectiveness

**Fig. 3:**
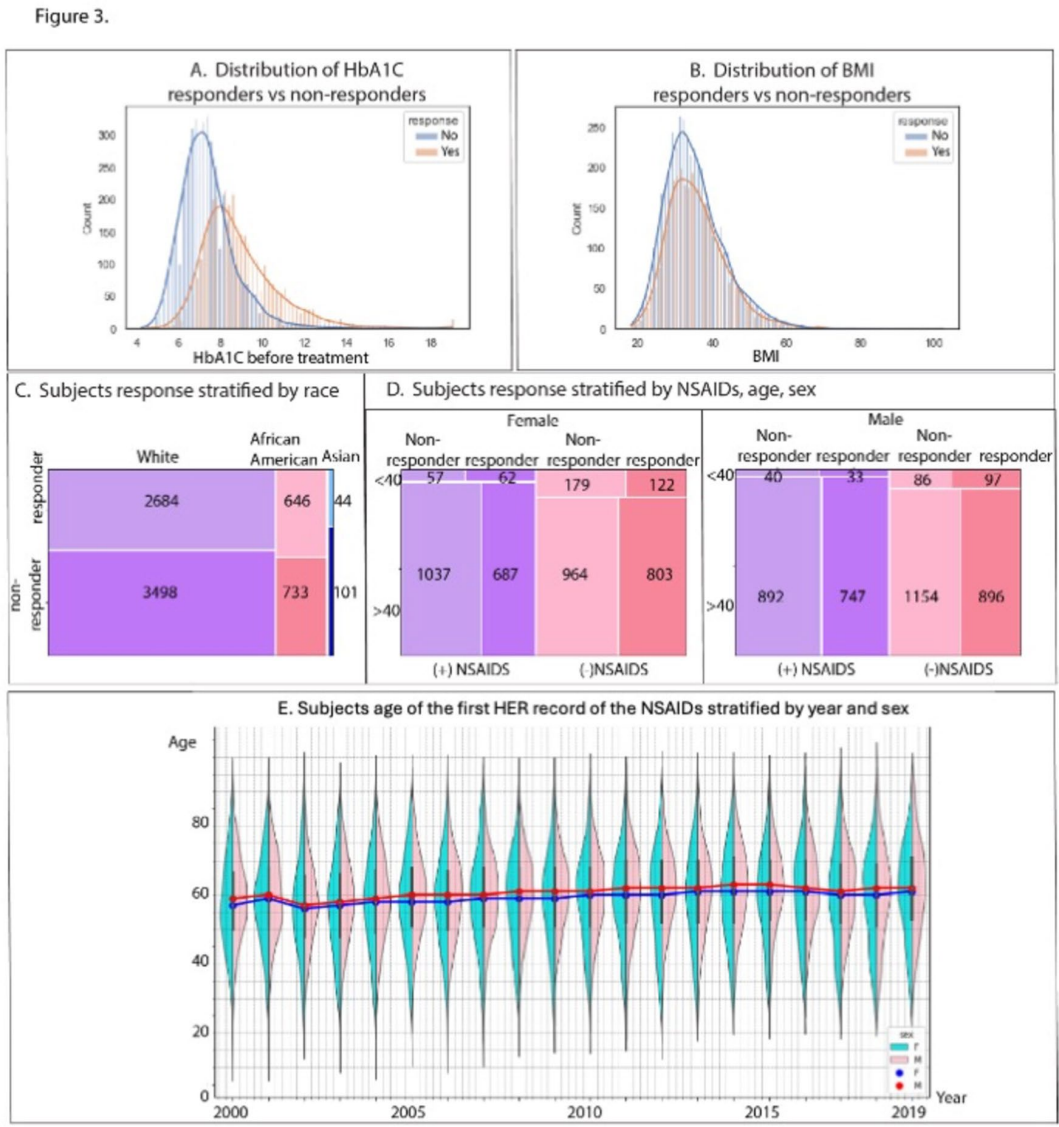
Distribution of responders and non-responders for several key features. **A**. Histogram showing range of HbA1C values of the study cohort pre-GLP-1 M treatment. Blue shading: HbA1C values for subjects that did not respond to GLP-1 M treatment. Orange shading: HbA1C values for subjects that responded positively to GLP-1 M treatment. Compared to the non-responder group, the curve for the responder group is right-shifted (toward higher pretreatment HbA1C values). **B**. Histogram showing BMI values for the study cohort pre-GLP-1 M treatment. Blue shading: BMI values for subjects that did not respond to GLP-1 M treatment. Orange shading: BMI values for subjects that responded positively to GLP-1 M treatment. Compared to the non-responder group, the curve for the responder group has fewer people with BMI lower than 40. **C**. Mosaic plot showing patient response to GLP-1 M treatment stratified by race. The numbers in the plots indicate the number of the subjects in each group. The top tiles indicate the number of the responders. The bottom tiles indicate the number of non-responders. Tiles from left to right are White, African American and Asian, respectively. **D**. Mosaic plots showing patient response stratified by NSAIDs usage, sex and age. The numbers in the plots indicate the number of the subjects in each group. Left plot: female subjects; Right plot: male subjects. In both plots, purple tiles indicate subjects treated with both GLP-1 M and NSAIDs, pink tiles indicate subject only treated with GLP-1 M. Top tiles: subjects who started GLP-1 M before or equal to 40 years old. Bottom tiles: subjects who started GLP-1 M after 40. **E**. Violin plots showing subjects age of the first EHR records of NSAIDs stratified by year and sex. Blue line: median age for Female, Red line, median age for Male

Univariate analysis was performed to explore features highly associated with GLP-1 M responsiveness without the complication of missing data. The results from the univariate analysis also aid in applying the model to other datasets in the future. This analysis revealed that pre-treatment HbA1C, fasting blood glucose, random blood glucose, and urine glucose were significantly higher in the responsive group (Table 3, Fig. 3A). Additionally, higher levels of total cholesterol, LDL, triglycerides, and lower HDL were observed in the responsive group, suggesting greater insulin resistance [15]. Elevated levels of serum glutamic pyruvic transaminase (SGPT, also as known as ALT), serum glutamic oxaloacetic transaminase (SGOT, also known as AST), urine albumin, and albumin/creatinine ratio were also noted, indicating potentially poorer kidney and liver function.

We also examined the association of other T2D medications with GLP-1 M response. Exogenous insulin administration correlated with enhanced responsiveness (Tables 4 and 5), with a significant decrease in HbA1C of 0.66 ± 1.81%. Combining GLP-1 M with metformin improved the response compared to non-insulin drugs (Tables 4 and 5). Conversely, the combination of GLP-1 M with thiazolidinediones was associated with a decreased response (Table 5), while sulfonylurea use showed no significant effect.

The ADA recommends using GLP-1 M for its additional cardiovascular and kidney benefits. Subjects with chronic kidney disease (CKD) had a reduced response to GLP-1 M treatment compared to those without CKD (Tables 4 and 5). Heart failure also correlated with a decreased response, whereas hypertension was associated with a slightly better response (Table 4). No significant differences were observed in subjects with weight loss surgeries, arthritis, or retinopathy.

In examining the potential effects of race on GLP-1 M responsiveness, subjects self-identifying as Asian showed a lower average reduction in HbA1C compared to non-Asian subjects, indicating potentially less benefit from GLP-1 M treatment for glucose control in Asian populations (Tables 4 and 5, Fig. 3C).

### Association of sex, age, and NSAIDs with responsiveness to GLP1-M treatment

NSAIDs, which are among the most commonly used medications for treating various medical conditions, have a complex relationship with glucose metabolism. While aspirin and sodium salicylate were historically used to treat diabetes and cardiovascular disease [16, 17], some newer NSAIDs have been associated with increased cardiometabolic risk. Some of the beneficial effects of first-generation COX inhibitors may have been due to decreased levels of prostaglandin E_2_ (PGE_2_), which acts through GPCRs, some of which are expressed in β cells along with the GLP-1R [18] and could modulate GLP-1R effects**.** Recent studies suggest that some NSAIDs directly inhibit DPP4, thus elevating endogenous GLP-1 [19]. However, our study found that subjects taking NSAIDs (a list of these medications is found in Supplemental Table 3) simultaneously with GLP-1 M collectively showed an overall weaker response to GLP-1 M treatment (Table 4, FDR = 5.16E-03). A similar pattern was observed when GLP-1 M was used together with other pain relievers such as acetaminophen and hydrocodone (Table 4, FDR = 6.76E-03). It was reported that NSAID usage varies with both sex and age [20, 21], and since sex hormones can affect various physiological responses, we divided the subjects into eight groups based on sex, age, and NSAID usage. Overall, our data suggest that females show reduced glucose-lowering benefit from GLP1-M treatment Table 4). However, as shown in Fig. 3D and Table 6, among females less than 40 years old, NSAID usage was associated with a greater reduction in HbA1C in response to GLP-1 M (0.82 ± 1.72) than no NSAID usage (0.28 ± 1.70, Bonferroni adjusted *p* = 8E-03). Interestingly, the relationship was reversed in females over age 40 (0.52 ± 1.44 without NSAIDs vs 0.36 ± 1.45 with NSAIDs, Bonferroni adjusted *p* = 2E-03). We did not observe any age-associated differences in GLP-1 M responsiveness in male subjects. There is also no significant difference in the percentage of subjects responding to GLP-1 M with or without NSAID usage (Fig. 3D). To understand whether there is a difference in NSAID usage between sexes, we compared the first records of NSAID usage in VUMC SD (Fig. 3E). We did not identify any significant difference in the distribution of NSAID usage. Given the widespread use of NSAIDs in clinical practice, our findings highlight the need for further investigation into the potential interactions between NSAIDs and GLP-1 M treatment.

**Table 6:** Response to GLP-1 M based on the combination of sex, age and NSAIDs

**Age**	**Sex**	**NSAID usage**	**Number of subjects**	**Decrease in HbA1c** **(mean** ± SD)	**Bonferroni-Adjusted p-Value**
Age < = 40	Male	No	183	0.78 ± 2.17	5.12E-1
		Yes	73	0.56 ± 2.42	
	Female	No	301	0.28 ± 1.70	**8E00-3**
		Yes	119	0.82 ± 1.72	
Age > 40	Male	No	1639	0.58 ± 1.66	2.42E-1
		Yes	2050	0.50 ± 1.54	
	Female	No	1767	0.52 ± 1.43	**2.00E-3**
		Yes	1724	0.36 ± 1.45	

Comparison of features of GLP-1M responders versus non-responders. Significant values are shown in bold in the right hand column

### Predictive model for GLP-1 M responsiveness

While univariate analysis offers valuable insights, our primary goal was to develop a predictive model for patient responsiveness to GLP-1 M treatment, irrespective of causal relationships. We next built a machine learning model to enhance prediction and further selected from the aforementioned features with less than 30% missing datapoints to build a prediction model for GLP-1 M responsiveness. Machine learning models with high complexity face the difficulty of generalizability. Thus, we used one of the simplest available models, logistic regression, as the benchmark. Our exploratory analysis revealed both positive and negative correlations between particular features (Supplemental Fig. 2). For example, chronic kidney disease was strongly correlated with heart failure, while it was negatively correlated with metformin usage. Random blood glucose was positively correlated with urine glucose, but negatively correlated with blood sodium. Therefore, to reduce model complexity further, we applied L1 regulation (also called Lasso). With L1 regulation, when two features are highly correlated, the coefficient for one of the features will be set to zero and is not considered in the final model.

**Fig. 4:**
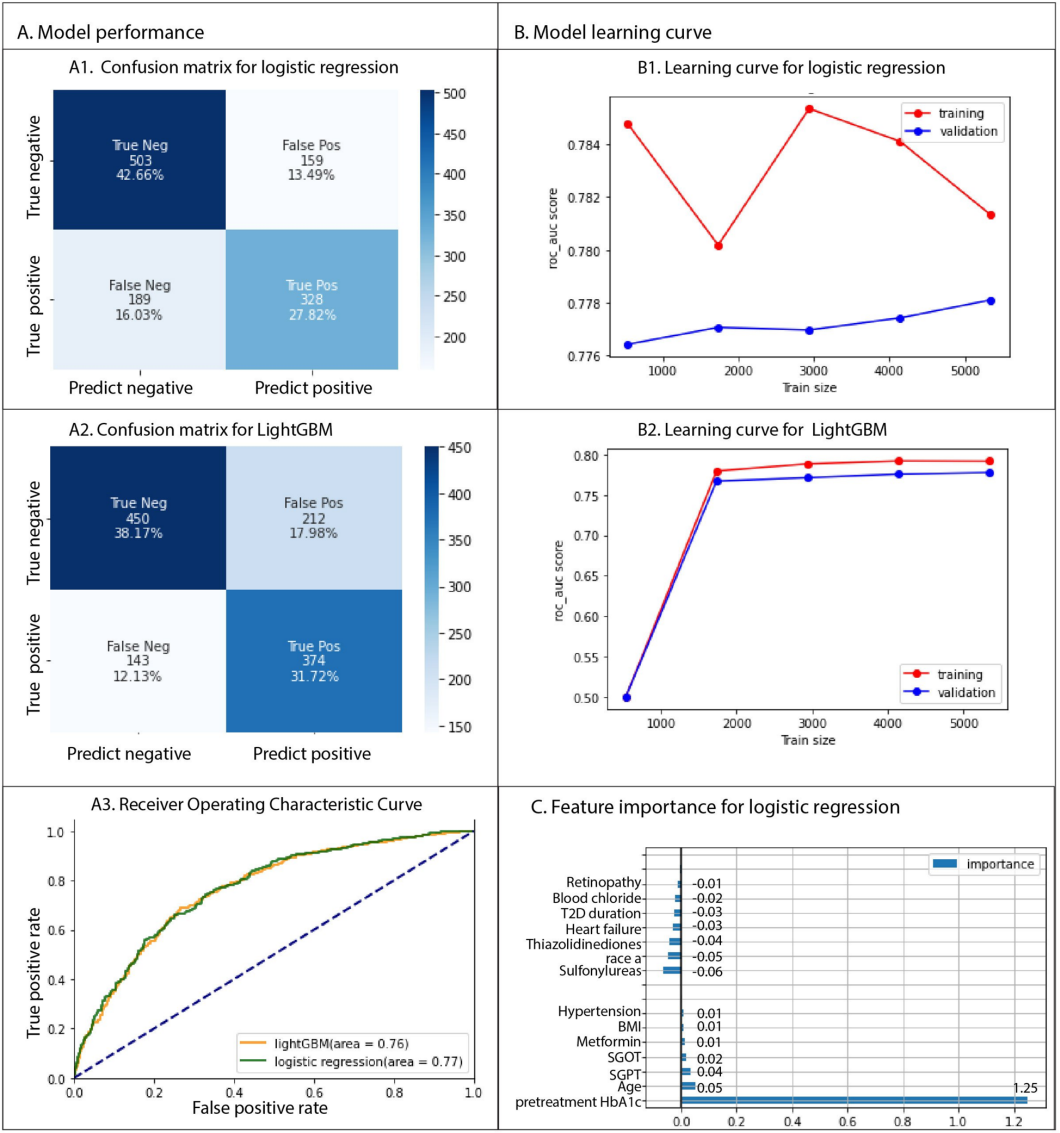
Assessment of model performance. **A**. Confusion matrices for: (**A**1) logistic regression and (**A**2) LightGBM. **A**3. ROC (Receiver Operating Characteristic) curve for logistic regression (green) and LightGBM (orange). The blue dotted line indicates where the false positive rate equals the true positive rate. **B**. Model learning curves for (**B**1) Logistic regression and (**B**2) LightGBM. Area under the ROC (auROC) curve was used to evaluate the model performance and is shown in the y-axis. C. Logisitic regression coefficients were plotted to show feature importance

We compared logistic regression with three different machine learning models using distinct algorithms: LightGBM, SVC, and ANNs. LightGBM is a gradient boosting framework using tree-based learning algorithms. SVC is used for classification tasks by finding the optimal hyperplane that maximizes the margin between different classes. ANNs consist of interconnected nodes (neurons) organized in layers: input, hidden, and output layers, capable of capturing complex patterns in data. The hyperparameters searched for all models are shown in Table 7. Model performance was assessed using a confusion matrix (Fig. 4A1, 4A2, Supplemental Fig. 3) and auROC (area under the Receiver Operating Characteristic curve; Fig. 4A3, Supplemental Fig. 3). As shown in Fig. 4A, all models performed similarly except SVC. Logistic regression achieved an auROC of 0.77 (Fig. 4A3), while LightGBM and ANN both achieved an auROC of 0.76 (Fig. 4A3, Supplemental Fig. 3). The performance of SVC was the worst among the models.

**Table 7:** Model hyperparameters and evaluation results

Model	Hyperparameter name	Hyperparameter values explored	Best value identified	Validation score (AUC)	Testing score (AUC)
Logistic regression	Fit intercept	True, False	False	0.79 ± 0.075	0.77
	C	0.0001, 0.001, 0.01, 0.002, 0.02	0.02		
LightGBM	Max depth	−1, 8, 32, 64	−1	0.79 ± 0.012	0.76
	Min data in leaf	50, 100, 200, 300, 400	400		
	Learning rate	0.01, 0.001, 0.1	0.01		
	Reg lambda	0.1, 0.5, 1, 10	0.5		
	Max bin	128, 256, 512, 1024	512		
	N estimators	200, 400, 800	400		
	Feature fraction	0.2,0.4, 0.8, 1.0	1.0		
	Num leaves	15, 31, 64,128,256	15		
	Reg alpha	0.5, 1, 10	1		
**Artificial Neural Network** **(ANN)**	Hidden layer size	(100,100,100), (50,100,50), (100,1)	(100,1)	0.67 ± 0.006	0.76
	Activation	relu, tanh, logistic	logistic		
	Alpha	0.0001, 0.05	0.05		
	Learning rate	constant,adaptive, invscaling	adaptive		
	Solver	adam	adam		
	Batch size	16,32,64,128	64		
Support Vector Classifier (SVC)	Kernel	Linear, poly, rbf, sigmoid	Sigmoid	0.269 ± 0.006	0.56
	Gamma	0.001, 0.0001, 0.1, 0.2, 0.005	0.1		
	Shrinking	False, True	False		

Model performance. Model hyperparameters and the final score. For each model, all combinations of the listed hyperparameters were tested using grid search. Hyperparameter names presented here follow the model parameter in the Sklearn (logistic regression) and LightGBM package

Since none of the other models tested outperformed logistic regression, we used LightGBM to explore whether increasing the sample size would further improve either model. As indicated in the learning curves for logistic regression (Fig. 4B), the training and validation sets would likely converge at around 0.78 if more samples were added. For LightGBM (Fig. 4B2), the learning curve has already reached a plateau, suggesting that increasing the sample size would not improve model performance.

As logistic regression proved to be the best model, the model coefficients are plotted in Fig. 4C. The full set of the coefficients and the odds ratio are in the Supplemental Table 6. Pre-treatment HbA1C, subject age, ALT, AST, BMI, metformin use, and having hypertension each positively contribute to predicted GLP-1 M responsiveness. These results are consistent with the univariant analysis results (Table 2). Interestingly, the model did not identify insulin usage as being predictive of treatment responsiveness. The use of insulin in subjects might be associated with features such as age and HbA1C level, so the coefficient for insulin was set to zero when L1 regulation was applied. Sulfonylurea or thiazolidinedione use, being of Asian descent, having heart failure, retinopathy, high blood chloride, and long T2D duration all negatively contributed to the predicted value of GLP-1 M effectiveness.

## Discussion

In this study, we used a de-identified patient EHR database to analyze the real-world response of T2D patients to GLP-1 M treatment and to predict which patients might benefit most from this class of T2D medications**.** Taken together**,** our model predicts that the best candidates for adding GLP-1 M as a second line T2D medication are: older age, non-Asian subjects, with short T2D duration that have elevated HbA1C, high BMI, hypertension, and no heart failure or retinopathy. A limitation of the current study is that it was performed using data from a single academic medical center with limited diversity in terms of patient population. The current results should be confirmed using datasets from different subject cohorts.

Our goal in the current study was to develop a predictive model that could guide the use of GLP-1 M medications for the treatment of T2D in the real world. To increase our power, the current study did not separately consider effects on GLP-1RA or DDP4i responsiveness. We specifically used common clinical factors that are available within current clinical practice to build our model, making it more feasible for use in real-world settings. As a starting point, we used the HbA1C 12 months before the decision point, which is typically available in clinical practice. Our model reveals clinical features that should be considered when deciding to initiate GLP-1 M combined therapy for the treatment of T2D, including age, race, T2D duration, HbA1C levels, BMI, hypertension, and the presence or absence of heart failure or retinopathy. By incorporating these factors into our model, we aim to provide clinicians with a more accurate and personalized tool for predicting which patients might benefit most from GLP-1 M treatment. Our model reveals clinical features that should be considered when deciding to initiate GLP-1 M combined therapy for the treatment of T2D. We want to emphasize that although these factors can be used to guide clinical decisions, they may not have a causal relationship with the response to GLP-1 M.

Some factors identified in this study are consistent with prior existing knowledge. Previous research showed that patients with shorter duration of T2D are more likely to benefit from GLP-1 M treatment [22]. Interestingly, univariate analysis did not identify differences in T2D duration between responder and non-responders (Table 3). However, in the final model, when considering all other factors, T2D duration was found to be negatively associated with GLP-1 M responsiveness (Fig. 4C), which is consistent with the previous study. The finding that hypertension and higher BMI positively contribute to predicting GLP-1 M responsiveness is consistent with the current approach of using GLP-1 M for protection of the cardiovascular system and promoting weight loss [3]. Consistent with these reports, recent research from one of our groups (Q.S.W.) further demonstrated that drugs targeting genes involved in the regulation of systolic blood pressure might be repurposed to treat diabetes [23].

The finding that high ALT and AST also predict a good response to GLP-1 M was not anticipated based on the literature, and seems counterintuitive. It is unlikely that impaired liver function enhances GLP-1 M effectiveness. Elevated ALT and AST might simply reflect fatty liver disease due to insulin resistance and worse severity of the underlying metabolic and physiological condition. In support of this hypothesis, we did identify a weak association between the level of C-peptide and the level of ALT (Pearson’s correlation coefficient = 0.30) and AST (Pearson’s correlation coefficient = 0.29). The popularity of GLP-1 M is partly due to its cardiovascular, kidney, and weight loss benefits. Our results suggest that although GLP-1 M have demonstrable protective effects on cardiovascular and kidney disease, for people who already have CKD and heart failure, such protection might come together with reduced benefit on lowering of blood glucose. On the other hand, for patients with hypertension, GLP-1 M have an enhanced blood glucose lowering effect.

The observation that differences in sex and age resulted in some subjects having better responsiveness to combined treatment with NSAIDs and GLP-1 M was also unexpected. One caveat of these studies is that many NSAIDs do not require a prescription and are one of the most commonly used over the counter medications. The current dataset is from the hospital records, which include self-reported information. Thus, we cannot rule out the possibility that some NSAID usage was not captured in the EHR/SD. Most likely, subjects listed as receiving NSAID treatment were experiencing chronic pain or inflammation. Subjects without NSAID treatment listing in the SD might include subjects that used NSAIDs only for a short time and did not inform their attending physician. Further research is required to validate the effects of NSAID usage and to understand the underlying mechanisms behind the interaction between NSAIDs and GLP-1 M. Several features, including NSAIDs and insulin usage, were ultimately not included in the final models. This was due to our decision to apply L1 regulation in order to obtain the simplest model for the purpose of helping clinicians determine which of their patients with T2D would most likely benefit from adding GLP-1 M as the second-line medication.

Our current analysis cannot exclude the possibility that the patient criteria for considering GLP-1 M treatment are generalizable for other T2D medications since we only considered GLP-1 M and did not compare it with responsiveness to other treatments. Future studies applying multi-label classification to a larger data set can help address this question. However, it has traditionally been believed that T2D drugs are more efficacious for subjects with higher HbA1C, partly due to the relief of the stress from glucotoxicity [24], and that is what we observed as well. In contrast, higher HbA1C has recently been linked to poor metformin responsiveness [25]. In our study, pretreatment HbA1C level was the dominant factor that affected GLP-1 M treatment outcome in the current analysis. Thus, for patients who have higher HbA1c at the time of diagnosis, it might be beneficial to use GLP1-M earlier in their course of treatment.

Our model also suggests that GLP-1 M treatment combined with sulfonylureas or thiazolidinediones has no added benefit and may actually decrease treatment effectiveness. In fact, both sulfonylureas and thiazolidinediones are negative predictors for GLP-1 M responsiveness. These associations are independent of the pre-treatment HbA1C for two reasons: 1. HbA1C is included in the model; 2. we did not observe any obvious differences in the pre-treatment HbA1C between subjects taking sulfonylureas and thiazolidinediones and those who were not taking these medications Previous studies have reported that combining sulfonylureas or thiazolidinediones with GLP-1 M is an effective treatment for T2D [26–29]. However, those studies did not compare the effectiveness of this drug combination with the use of GLP-1 M alone. For example, in a previous study, the effectiveness of dulaglutide combined with pioglitazone was compared with that of pioglitazone and metformin [29]. In our analysis, we compared the effectiveness of GLP-1 M treatment alone with that of the combination of GLP-1 M treatment with other medications. Interestingly, we only observed the negative effects of thiazolidinediones and sulfonylureas on GLP-1 M effectiveness in our final predictive model, which was built using all the features shown in Supplemental Fig. 2. Our univariate analysis did not detect any association. Therefore, further study is required to fully understand the combined effect of these drugs on improving HbA1C**.**

Machine learning and artificial intelligence have been successfully used in various fields. Although machine learning models often appear to be a black box, having such a prediction model would be extremely beneficial for real-world applications and patient treatment decisions. Our best prediction model was the logistic regression model with a 0.77 auROC. Complex models such as LightGBM and artificial neural network all performed similarly to logistic regression. This result is not unprecedented; in a recent study predicting patient survival after heart transplantation, all models tested demonstrated similar performance to logistic regression [30]. The authors hypothesized that the quality of the clinical datasets might limit the application of the machine learning techniques. In addition to data quality, in our experience one limitation comes from the inherent bias within healthcare data sets. A typical patient has a limited number of standard-of-care lab tests commonly ordered by their attending physician. Thus, the models used here had no opportunity to explore more rare features which might have as yet un-identified relationships with GLP-1 M treatment. Since it is impossible to test all the lab values for a patient, a combination of genomic information with clinical information might be the best way to identify the individuals that will benefit most from GLP-1 M treatment. In the future, approaches such as this may help to define physiologic and mechanistic causes for the differences in GLP-1 M responsiveness. 

In conclusion, this study demonstrates that a predictive model based on commonly available clinical features can guide the use of GLP-1 M medications in T2D treatment. By identifying key factors such as age, race, T2D duration, HbA1C levels, BMI, hypertension, and the presence of other conditions like heart failure or retinopathy, our model provides a personalized tool for clinicians to make informed treatment decisions. Future research incorporating patients’ genomic information are expected to build better model.

## Supplementary Information

Supplementary Material 1.

Supplementary Material 2.

Supplementary Material 3.

Supplementary Material 4.

Supplementary Material 5.

Supplementary Material 6.

Supplementary Material 7.

Supplementary Material 8.

Supplementary Material 9.

Supplementary Material 10.

Supplementary Material 11.

## Data Availability

All the data in this study are from the VUMC SD database. Restriction and institutional approval are required for data access. Therefore, the data for this study are not publicly available. The original code is in a Jupyter notebook with the data embedded. Thus, they are not publicly available to protect the data used in this study.
