# Supplementary figures and images for: Predicting responsiveness to GLP-1 pathway drugs using real-world data
Source: BMC Endocr Disord. 2024 Dec 18;24:269. doi: 10.1186/s12902-024-01798-9 (PMC11654408; doi:10.1186/s12902-024-01798-9)

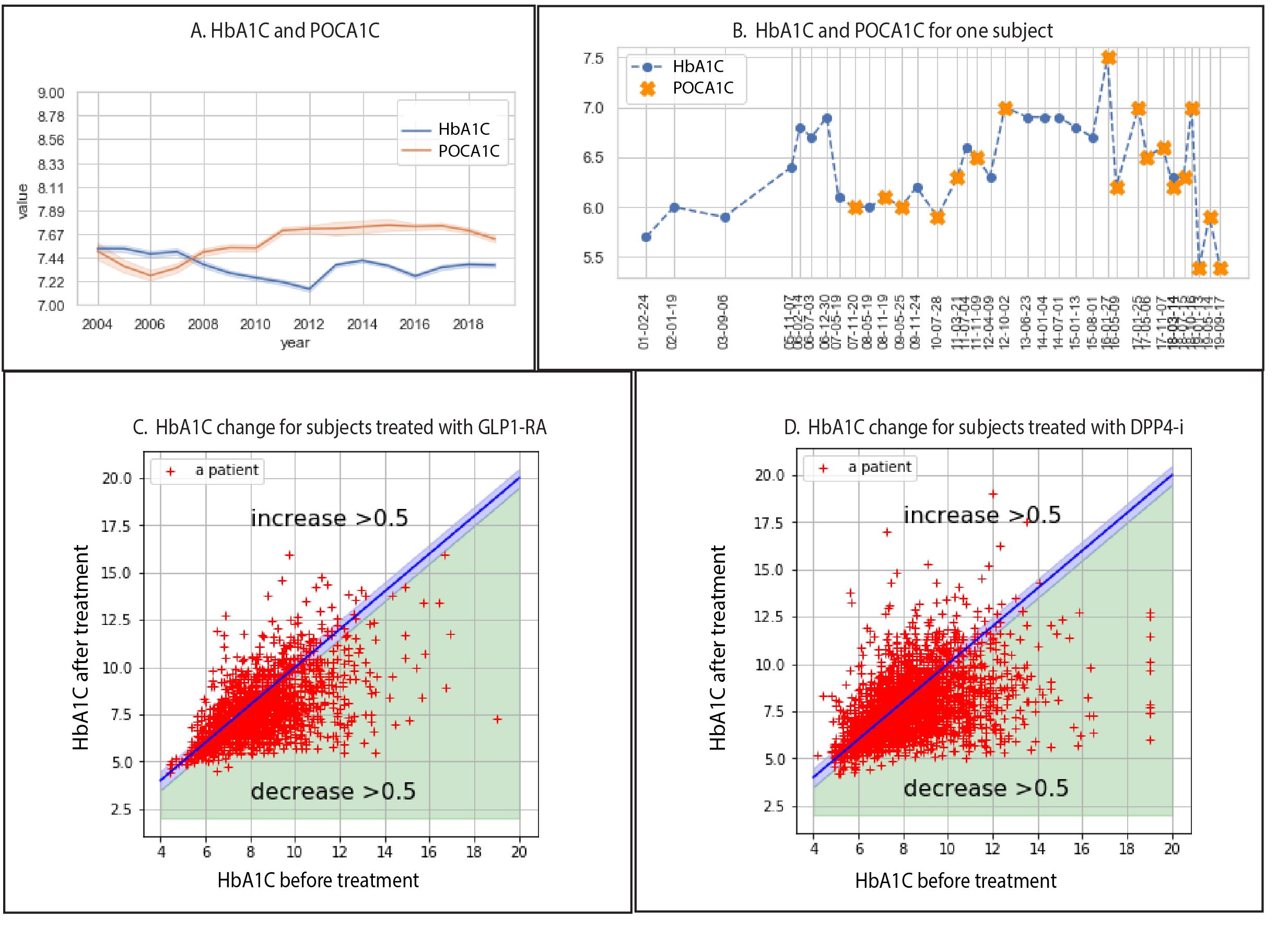

Supplement: Supplementary file 1 — Supplementary Material 1. [file 12902_2024_1798_MOESM1_ESM.jpg]

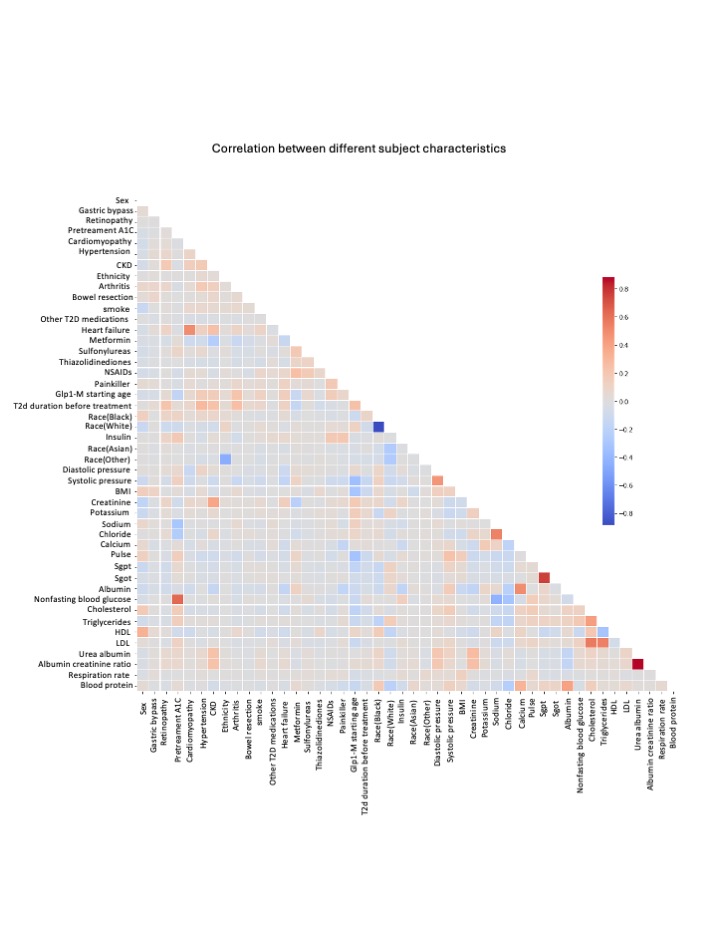

Supplement: Supplementary file 2 — Supplementary Material 2. [file 12902_2024_1798_MOESM2_ESM.jpeg]
