# Supplementary material for: Predicting responsiveness to GLP-1 pathway drugs using real-world data
Source: BMC Endocr Disord. 2024 Dec 18;24:269. doi: 10.1186/s12902-024-01798-9 (PMC11654408; doi:10.1186/s12902-024-01798-9)
Supplement: Supplementary file 7 — Supplementary Material 7. [file 12902_2024_1798_MOESM7_ESM.docx]

**Supplemental Table 5A. Summary statistics for training and testing data sets.** **(binary variables)**

|  | **Training/Validation**  **case number (percentage)** | **Testing**  **case number (percentage)** |
| --- | --- | --- |
| **Total** | 6677 | 1179 |
| **Female** | 3325 (49.80%) | 586(49.7%) |
| **Not Hispanic** | 6485(97.12%) | 1150(97.54%) |
| **Africa American** | 1179(17.66%) | 207(17.56%) |
| **White** | 5255(78.70%) | 938(79.56%) |
| **Asian** | 132(1.97%) | 17(1.44%) |
| **Other** | 129(1.93%) | 18(1.52%) |
| **Chronic kidney disease** | 921(13.79%) | 148(12.55%) |
| **Cardiomyopathy** | 292(4.37%) | 49(4.16%) |
| **Heart failure** | 564(8.45%) | 90(7.633%) |
| **Hypertension** | 4708(70.51%) | 858(72.77%) |
| **Arthritis** | 1253(18.76%) | 194(16.45%) |
| **Gastric bypass** | 121(1.81%) | 27(2.29%) |
| **Bowel resection** | 70 (1.04%) | 12(1.02%) |
| **Retinopathy** | 283 (4.24%) | 50(4.24%) |
| **Insulin** | 2031(25.78%) | 310(26.29%) |
| **Metformin** | 4034(60.41%) | 700(59.37%) |
| **Sulfonylureas** | 2587(38.74%) | 454(38.51%) |
| **Thiazolidinediones** | 654(9.79%) | 119(10.09%) |
| **NSAIDs** | 3364(50.38%) | 602(51.06%) |
| **Painkiller** | 1300(19.46%) | 242(20..52%) |
| **Other T2D Medication** | 412(6.17%) | 76(6.45%) |
| **Smoking** | 2443(36.59%) | 445(37.74%) |

|  | **Training/Validation set**  **Mean +/- std** | **Testing set**  **Mean +/- std** |
| --- | --- | --- |
| **HbA1C before treatment** | 8.01$\pm$1.72 | 8.02$\pm$1.69 |
| **HDL** | 43.35$\pm$12.63 | 43.26$\pm12.62$ |
| **LDL** | 105.67$\pm75.20$ | 103.34$\pm70.27$ |
| **Triglycerides** | 208.79$\pm157.20$ | 214.69$\pm187.66$ |
| **Total cholesterol** | 176.20$\pm45.13$ | 177.88$\pm49.98$ |
| **SGOT(AST)** | 32.86$\pm26.60$ | 29.08$\pm17.45$ |
| **SGPT(ACT)** | 29.27$\pm20.50$ | 32.41$\pm$21.50 |
| **Random glucose** | 174.85$\pm72.11$ | 175.62$\pm72.25$ |
| **SBP** | 76.14$\pm9.99$ | 76.03$\pm$10.00 |
| **DBP** | 132.89$\pm14.68$ | 132.92$\pm$15.00 |
| **BMI** | 35.36$\pm8.06$ | 35.95$\pm$8.14 |
| **Blood albumin** | 4.19$\pm0.36$ | 4.17$\pm$0.38 |
| **Albumin creatinine ratio** | 73.86$\pm264.26$ | 65.54$\pm$270.98 |
| **Urine albumin** | 74.63$\pm257.70$ | 70.18$\pm$251.92 |
| **Total blood protein** | 7.25$\pm0.51$ | 7.25$\pm$0.51 |
| **Blood calcium** | 9.53$\pm0.48$ | 9.53$\pm$0.47 |
| **Blood chloride** | 102.34$\pm3.35$ | 102.30$\pm$3.42 |
| **Blood potassium** | 4.23$\pm0.42$ | 4.26$\pm$0.43 |
| **pulse** | 80.04$\pm12.01$ | 80.01$\pm$12.36 |
| **respiration rate** | 17.46$\pm3.37$ | 17.60$\pm$3.72 |
| **Blood sodium** | 138.54$\pm2.58$ | 138.54$\pm2.66$ |
| **Age** | 57.30$\pm11.92$ | 57.30$\pm$11.80 |
| **T2D duration** | 3.36$\pm3.96$ | 3.39$\pm$3.89 |

**Supplemental Table 5B. Summary statistics for training and testing data sets.** **(continuous variables)**
