# Supplementary material for: Predicting responsiveness to GLP-1 pathway drugs using real-world data
Source: BMC Endocr Disord. 2024 Dec 18;24:269. doi: 10.1186/s12902-024-01798-9 (PMC11654408; doi:10.1186/s12902-024-01798-9)
Supplement: Supplementary file 9 — Supplementary Material 9. [file 12902_2024_1798_MOESM9_ESM.docx]

**Supplemental Table 7A. Summary statistics of the features used in this study (continuous features).**

|  | **Case number** | **mean**±**std** |
| --- | --- | --- |
| **HbA1c decrease** | 7856 | 0.50±1.57 |
| **HbA1C before treatment** | 7856 | 8.01±1.72 |
| **HDL** | 5635 | 43.35±12.63 |
| **LDL** | 5279 | 105.33±74.50 |
| **Triglycerides** | 5652 | 209.66±162.08 |
| **Total cholesterol** | 5713 | 176.45±45.89 |
| **SGOT(AST)** | 6480 | 29.24±20.07 |
| **SGPT(ACT)** | 6546 | 32.80±25.90 |
| **C -peptide** | 324 | 4.05±3.22 |
| **Vitamin_B12** | 1094 | 747.69±926.74 |
| **Blood creatinine** | 7191 | 1.07±0.70 |
| **Fasting blood glucose** | 1215 | 164.56±62.13 |
| **Random glucose** | 5922 | 174.97±72.13 |
| **Urine glucose** | 1745 | 30.97±209.83 |
| **SBP** | 7769 | 76.12±9.99 |
| **DBP** | 7769 | 132.90±14.72 |
| **BMI** | 7705 | 35.45±8.07 |
| **Blood albumin** | 6168 | 4.19±0.36 |
| **Albumin /creatinine ratio** | 4019 | 72.63±265.25 |
| **Urine albumin** | 4021 | 73.97±256.83 |
| **Bicarbonate** | 399 | 25.20±5.29 |
| **Total blood protein** | 3478 | 7.25±0.52 |
| **Blood calcium** | 7087 | 9.53±0.48 |
| **Blood chloride** | 7089 | 102.34±3.36 |
| **Vitamin D25OH** | 1215 | 29.93±14.05 |
| **Whole blood lactate** | 265 | 1.50±0.77 |
| **O2_saturation** | 256 | 97.68±3.10 |
| **Blood potassium** | 7103 | 4.23±0.42 |
| **Pulse** | 6895 | 80.03±12.06 |
| **Respiration rate** | 3838 | 17.48±3.43 |
| **Blood sodium** | 7097 | 138.54±2.59 |
| **Age** | 7856 | 57.30±11.90 |
| **T2D duration** | 7856 | 3.36±3.95 |

**Supplemental Table 7B. Summary statistics of the features used in this study (discrete features).**

|  | **Case Number** |
| --- | --- |
| **Total** | 7856 |
| **Female** | 3911(49.78%) |
| **Not Hispanic** | 7635(97.19%) |
| **Africa American** | 1386(17.64%) |
| **White** | 6193(78.83%) |
| **Asian** | 149(1.90%) |
| **Other** | 147(1.87%) |
| **Chronic kidney disease** | 1069(13.61%) |
| **Cardiomyopathy** | 341(4.34%) |
| **Heart failure** | 654(8.32%) |
| **Hypertension** | 5566(70.85%) |
| **Arthritis** | 1447(18.42%) |
| **Gastric bypass** | 148(1.88%) |
| **Bowel resection** | 82(1.04%) |
| **Retinopathy** | 333(4.24%) |
| **Insulin** | 2031(25.85%) |
| **Metformin** | 4734(60.21%) |
| **Sulfonylureas** | 3041(38.71%) |
| **Thiazolidinediones** | 773(9.84%) |
| **NSAIDs** | 3966(50.48%) |
| **Painkiller** | 1542(19.63%) |
| **Other T2D medication** | 488(6.21%) |
| **Smoking** | 2888(36.76%) |

**Supplemental Table 7C. The distribution of Patients HbA1C levels before GLP1 treatment**

| HbA1C range | Percentage |
| --- | --- |
| HbA1C <7.0 | 27.1% |
| 7.0 <= HbA1C <10 | 61.4% |
| HbA1C >10 | 11.4% |

**Legend for Supplemental Table 7: Summary statistics of the features used in this study** A. Continuous features. B. Binary features. C. The distribution of HbA1C before GLP1 treatment
