# Supplementary material for: Predicting responsiveness to GLP-1 pathway drugs using real-world data
Source: BMC Endocr Disord. 2024 Dec 18;24:269. doi: 10.1186/s12902-024-01798-9 (PMC11654408; doi:10.1186/s12902-024-01798-9)
Supplement: Supplementary file 10 — Supplementary Material 10. [file 12902_2024_1798_MOESM10_ESM.docx]

**Supplemental Table 8A. Differences in responder vs non-responder subjects (binary variables)**

|  |  | **# of responders** | **FDR** |
| --- | --- | --- | --- |
| **SEX** | **Female** | 1674(42.8%) | 9.70E-02 |
|  | **Male** | 1773(44.9%) |  |
| **Ethnicity** | **Not Hispanic** | 3349(43.8%) | 9.42E-01 |
|  | **Hispanic** | 98(44.3%) |  |
| **Race** | **Africa American** | 648(46.8%) | **3.41E-02** |
|  | **Non-Africa American** | 2799(43.3%) |  |
|  | **White** | 2688(43.4%) | 1.60E-01 |
|  | **Non-White** | 759(45.6%) |  |
|  | **Asian** | 46(30.8%) | **4.02E-03** |
|  | **Non-Asian** | 3401(44.1%) |  |
|  | **Other** | 74(50.3%) | 1.88E-01 |
|  | **Non-Other** | 3373(43.7%) |  |
| **Chronic kidney disease** | **Yes** | 425(39.7%) | **8.05E-03** |
|  | **No** | 3022(44.5%) |  |
| **Cardiomyopathy** | **Yes** | 137(40.1%) | 2.35E-01 |
|  | **No** | 3310(44.0%) |  |
| **Heart failure** | **Yes** | 251(38.3%) | **7.58E-03** |
|  | **No** | 3196(44.3%) |  |
| **Hypertension** | **Yes** | 2494(44.8%) | **1.99E-02** |
|  | **No** | 953(41.6%) |  |
| **Arthritis** | **Yes** | 609(42.1%) | 1.91E-01 |
|  | **No** | 2838(44.3%) |  |
| **Gastric bypass** | **Yes** | 69(46.6%) | 5.83E-01 |
|  | **No** | 3378(43.8%) |  |
| **Bowel resection** | **Yes** | 33(40.2%) | 6.01E-01 |
|  | **No** | 3414(43.9%) |  |
| **Retinopathy** | **Yes** | 137(41.1%) | 3.71E-01 |
|  | **No** | 3310(44.0%) |  |
| **insulin** | **Yes** | 1017(50.1%) | **6.04E-10** |
|  | **No** | 2430(41.7%) |  |
| **Metformin** | **Yes** | 2130(44.5%) | **2.80E-02** |
|  | **No** | 1317(42.2%) |  |
| **Sulfonylureas** | **Yes** | 1359(44.7%) | 3.29E-01 |
|  | **No** | 2088(43.36%) |  |
| **Thiazolidinediones** | **Yes** | 289(37.3%) | **3.81E-04** |
|  | **No** | 3158(44.6%) |  |

**Supplemental Table 8B. Response to GLP-1M based on the combination of sex, age and NSAIDs**

| **Age** | **Sex** | **NSAIDs** | **Case number** | **Average a1c decrease** | **Adjusted p** |
| --- | --- | --- | --- | --- | --- |
| Age <= 40 | Male | No | 183 | 0.78±2.17 | 5.12E-1 |
|  |  | Yes | 73 | 0.56±2.42 |  |
|  | Female | No | 301 | 0.28±1.70 | **8E00-3** |
|  |  | Yes | 119 | 0.82±1.72 |  |
| Age > 40 | Male | No | 1639 | 0.58±1.66 | 2.42E-1 |
|  |  | Yes | 2050 | 0.50±1.54 |  |
|  | Female | No | 1767 | 0.52±1.43 | **200E-3** |
|  |  | Yes | 1724 | 0.36±1.45 |  |
